# Integrating transcriptomic and proteomic analyses reveals impaired carbohydrate metabolism in tobacco cytoplasmic male sterility

**DOI:** 10.3389/fpls.2025.1695628

**Published:** 2025-11-19

**Authors:** Chaoliang Hou, Yijie Liu, Yingjun Zhang, Ziqi Wang, Yixiang Cao, Can Tan, Qin Li, Zuohua Ren, Wei Zhou

**Affiliations:** 1 Hunan Provincial Engineering and Technology Research Center for Agricultural Big Data Analysis and Decision-Making, Hunan Agricultural University, Changsha, China; 2 Biotechnology and Data Science Innovation Research Institute, Huazhi Rice Bio-Tech Co., Ltd., Changsha, China

**Keywords:** CMS, carbohydrate metabolism, stamen development, tobacco, transcriptome-proteome, transcription factor

## Abstract

Cytoplasmic male sterility (CMS) is a vital tool for exploiting heterosis to enhance agricultural productivity. However, the genetic and molecular mechanisms of CMS in tobacco remain largely unclear. In this study, we used tobacco Gexin 3 CMS lines and their corresponding homozygous maintainer lines to systematically analyse the regulatory networks underlying CMS using transcriptomic, proteomic and morphological techniques. Morphological observations revealed premature stamen degeneration in CMS lines, resulting in failed self-pollination. Integrated transcriptomic and proteomic analyses identified 5,024 differentially expressed genes (DEGs) and 159 differentially expressed proteins (DEPs). Pathway enrichment analysis revealed that carbohydrate metabolism is a critical process in CMS. Several key enzymes, including hexokinase, pyrophosphate-fructose 6-phosphate 1-phosphotransferase and glyceraldehyde-3-phosphate dehydrogenase, were significantly downregulated at both the transcript and protein levels in the CMS lines. Reduced expression or functional impairment of these enzymes likely restricts the supply of substrates and ATP, thereby impairing floral and pollen development and ultimately reducing fertility. Network analysis identified several transcription factors as potential regulators of carbohydrate metabolism genes involved in floral organ development, including a CCCH-type zinc finger protein, an ethylene-responsive factor RAP2–4 and a LOB domain-containing protein. Taken together, these findings shed new light on the molecular basis of CMS in tobacco and lay the groundwork for exploring CMS regulatory networks in other crop species.

## 1 Introduction

Cytoplasmic male sterility (CMS) is a maternally inherited trait governed by the cytoplasmic genome, characterized by the inability of plants to produce functional pollen. This phenomenon has been observed in over 150 species of higher plants (Zheng et al., 2018). Although CMS is caused by an interaction between the cytoplasm and single nuclear recovery gene, it can be restored by the dominant allele of the nuclear fertility locus, making it extremely valuable for improving agricultural productivity (Yuan et al., 2018). Pollen development is a highly complex process primarily regulated by nuclear genes and transcription factors (TFs), and CMS genes may affect the expression of these nuclear genes (Liu et al., 2020). Thus, CMS is also a valuable resource for studying nuclear-cytoplasmic interactions. A comprehensive investigation of the molecular genetic mechanisms of CMS holds significant theoretical value and application prospects for crop breeding.

Carbohydrate metabolism forms the basis of cellular energy homeostasis and plays a critical role in supporting high-energy-demanding processes such as anther and pollen development (Yang et al., 2017). Disruption of carbohydrate metabolic pathways reduces the supply of substrates for glycolysis and the tricarboxylic acid (TCA) cycle, leading to insufficient ATP production and an imbalance of reactive oxygen species (ROS) (Ma et al., 2022). Such energy deficiency and oxidative stress are often closely associated with abnormal tapetum degradation and pollen abortion (Mingyue et al., 2025). Furthermore, transcriptional regulation is essential for coordinating carbohydrate metabolism with anther development, and disruption of these regulatory networks can further impair energy metabolism and pollen formation, ultimately resulting in male sterility (Liu S. et al., 2021).

Transcriptomic and proteomic approaches have been extensively employed to investigate the molecular mechanisms of CMS in higher plants such as *Brassica napus*, *Capsicum annuum*, *Gossypium hirsutum*, *Zea mays* and *Triticum* sp*elta*, among others (Wang et al., 2023). These analyses have revealed that CMS-related genes in the *Brassica napus* Shaan2A line primarily regulate carbohydrate and energy metabolism (Ning et al., 2019). In S-CMS *Triticum* sp*elta*, certain proteins related to carbohydrate metabolism are expressed abnomally, and key enzymes and genes involved are significantly down-regulated (Ge et al., 2023). Similarly, carbohydrate metabolism is central to the sterility process in the male sterile *Gossypium hirsutum* line 1355A (Wu et al., 2019). Carbohydrate metabolism is known to be the fundamental pathway for energy and nutrient supply essential for plant growth and development (Liu et al., 2024). Increasing evidence suggests a close association between carbohydrates and male sterility (Sun et al., 2022). For instance, sucrose transport is crucial for pollen development, serving both as a prerequisite for starch biosynthesis and as a key regulator of downstream metabolic processes (Tian et al., 2021). Insufficient sugar supply results in diminished ATP synthesis and energy deprivation, ultimately inducing male sterility (Yang et al., 2017). Sucrose hydrolysis is also essential for pollen development (Ruan et al., 2010), and starch accumulation is vital for fertility (Hsieh et al., 2023; Zhang et al., 2015). Three TFs play essential roles in rice pollen maturation and starch accumulation. The MYB TFs *OsCSA* and *OsCSA2* regulate anther and pollen development under short-day and long-day conditions, respectively, by promoting the expression of genes involved in sugar metabolism and transport, thereby facilitating long-distance sugar partitioning for pollen maturation (Zhang et al., 2010; Wang et al., 2021). Knockdown of *OsBZR1* (encoding a BR-signaling TF) expression causes defective pollen maturation with less starch accumulation (Zhu et al., 2015). These findings underscore the critical role of carbohydrates and their metabolism in CMS.

Despite these advances, the mechanisms of CMS in major crops such as tobacco remain understood (Bohra et al., 2025). In tobacco, integrative transcriptomic and proteomic analyses have also been applied to explore CMS mechanisms. The CMS line Yunyan 87 (MSYY87) and its maintainer line revealed that genes/proteins involved in lipid transport/binding and phenylpropane metabolism were significantly down-regulated, and several key candidates such as *β-GLU*, *4CL*, and *bHLHs* were proposed to play critical roles in energy metabolism, anther wall formation, and tapetum degradation (Mo et al., 2023). However, the molecular basis of carbohydrate metabolism in tobacco CMS remains largely unresolved, which is the primary focus of the present study. Therefore, this study aims to systematically elucidate the critical role of carbohydrates and their metabolic processes in tobacco male sterility based on the transcriptome and proteome of sterile and homozygous maintainer lines. This research will lay a strategic theoretical foundation for a deeper understanding of the molecular mechanisms underlying tobacco CMS and provide new insights for crop genetic improvement and hybrid advantage utilization.

## 2 Materials and methods

### 2.1 Collection of experimental materials and flower bud samples

Fresh flower buds were collected from the CMS line MSGX3 and its homozygous maintainer line GX3, which were cultivated in the experimental field of Jiangxi Agricultural University, Xinjian District, Nanchang City, Jiangxi Province, China (28°45′51″N, 115°50′28″E). The cytoplasm of GX3 originated from the fluecured tobacco cultivar, while that of MSGX3 was derived from the male sterile mutant of the Jiangxi suncured tobacco cultivar “Tiegu”. After more than ten generations of backcrossing, the sterility of MSGX3 was stabilized, and its nuclear background was made consistent with that of GX3.

To ensure the representativeness of the samples and the accuracy of the analysis, flower buds, including sepals and petals, were selected at five developmental stages: 1.5 cm, 2 cm, 2.5 cm, 3 cm, and 3.5 cm, following previous studies on tobacco flower development (Koltunow et al., 1990). The longitudinal diameter of the flower buds was measured using vernier calipers, and the morphological characteristics of their stamens were observed using a Motic SM7 stereomicroscope (Motic Scientiffc, Hong Kong, China) equipped with a Canon digital camera. Simultaneously, 2 cm flower buds were rapidly frozen in liquid nitrogen and stored at -80°C for subsequent transcriptome and proteome sequencing.

### 2.2 RNA sequencing and tandem mass tag analysis

Total RNA was extracted from tobacco buds using the TRIzol^®^ Reagent Kit, and sequencing libraries were constructed using the TruSeq™ RNA Sample Prep Kit. The libraries were sequenced in paired-end mode on the Illumina HiSeq X Ten platform. Low-quality reads and adapter sequences were removed using Trimmomatic, and the filtered reads were aligned to the tobacco reference genome (Ntab-TN90) using HISAT2. Gene expression levels were quantified using featureCounts and normalized using the Fragments per kilobase of transcript per million mapped reads (FPKM) method. TMT-based proteomic analysis was conducted in this study. Total protein extraction was carried out using a standard protocol. Protein concentrations were measured using the Bradford protein quantification kit, and sample proteins were labeled with TMT tags. Chromatography was conducted using an L-3000 high-performance liquid chromatography (HPLC) system, and peptides were separated by liquid chromatography for electrospray ionization (ESI) mass spectrometry in a Q Exactive™ HF-X instrument. Database searches and protein quantification were conducted using Proteome Discoverer 2.4 (PD2.4, Thermo Fisher Scientific).

### 2.3 Data analysis and biofunctional annotation

For differential expression analysis, transcriptome quantification results were statistically analysed using DESeq2, and proteome quantification results were assessed for intergroup differences using t-test. To integrate the transcriptomic and proteomic data, a nine-quadrant plot analysis was performed based on the expression matrices of the two groups. |log_2_ fold change| ≥ 2 was adopted as the threshold criterion for the transcriptome, and the threshold value for the proteome was FC ≥ 1.5 or FC ≤ 2/3 in order to assess the correlation of the expression between different omics data. Subsequently, gene ontology (GO) functional enrichment analysis was performed on the screened DEGs and DEPs using the enrichGO function in the clusterProfiler software package, and Kyoto Encyclopedia of Genomes (KEGG) pathway enrichment analysis was performed using the KOBAS 2.0 platform.

### 2.4 Interactive analysis

Interactions between transcription factors and carbohydrate metabolism-related proteins were analyzed using the STRING database. Target gene sequences were aligned with reference protein sequences using BLASTx (v2.2.28), and interaction networks were constructed based on known tobacco protein interactions.

### 2.5 Measurement of metabolism-related indices

ATP and pyruvate contents in tobacco buds were measured using an ATP assay kit (AKOP004U, Boxbio) and a pyruvate assay kit (AKAC002C, Boxbio). Concentrations of sucrose, fructose, starch, and soluble sugars were measured using the Plant Sucrose Content Assay Kit (AKPL006C, Boxbio), Fructose Content Assay Kit (AKPL007C, Boxbio), Starch Content Assay Kit (AKSU015C, Boxbio), and Soluble Sugar Content Assay Kit (AKPL008C, Boxbio), respectively. All procedures and calculations were performed according to the manufacturer’s instructions.

### 2.6 Real-time quantitative PCR assay

Based on transcriptomic and proteomic analysis results, candidate CMS genes were selected for RT-qPCR detection to assess gene expression levels. Primer design was performed using the Primer Premier 5 tool. RT-qPCR reactions were performed with cDNA as a template with the Takara SYBR Premix Ex Taq Kit. Each reaction was completed on an Applied Biosystems 7500 Real-Time PCR System (Life Technologies, Beverly, MA, USA). The amplification program included a 30-second predenaturation step at 95°C, followed by 40 cycles (each cycle consisting of 5 seconds denaturation at 95°C and 30 seconds annealing/extension at 60°C). Melt curve analysis was performed postamplification to verify primer specificity. **Supplementary Table S1** lists the genes and corresponding primers used for RT-qPCR detection. Relative gene expression levels were calculated using the 2^-ΔΔCt method.

## 3 Results

### 3.1 Analysis of differences in morphological characteristics of stamens

**Figure 1:**
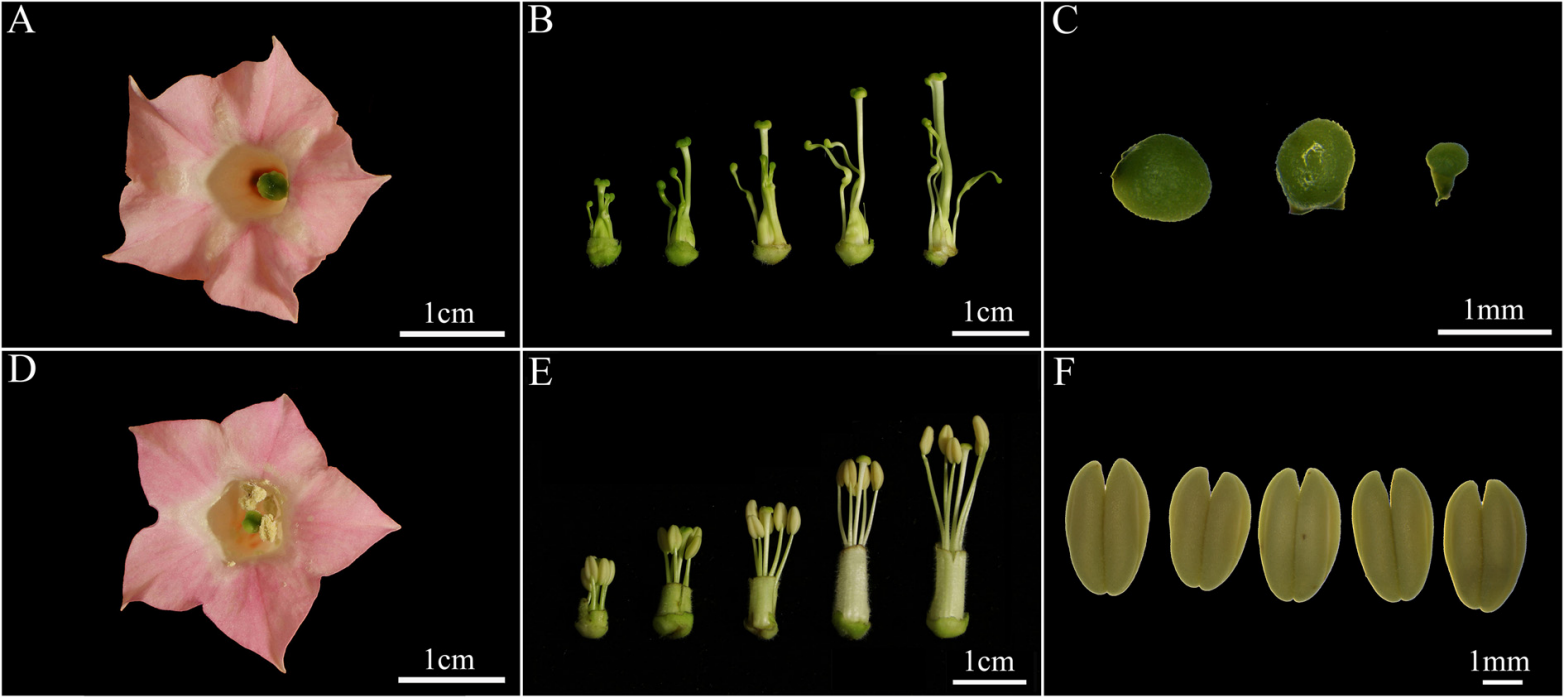
Morphological characteristics of flowering organs from CMS lines **(A-C)** and maintainer lines **(D-F)** of the GX3 cultivar. **(A, D)** represented the appearance of mature flowers, **(B, E)** represented the buds at various periods, and **(C, F)** represented the morphology of anthers at the pre-flowering stage.

No significant differences were observed in the morphology of floral organs such as petals and pistils between the sterile and maintainer lines; however, the morphology of the stamens exhibited significant differences (**Figures 1A, D**). The morphology of the stamens of the maintainer lines was normal, characterised by five intact anthers which were located near or above the stigma; the anthers were uniformly yellowish, uniform in size and could dehisce normally when the flower opened (**Figures 1E, F**; **Supplementary Figure S1**). In contrast, the sterile lines had severely degenerated stamens, short and twisted filaments, and uniform green anthers similar in shape to the stigma; the shape and colour of the anthers in the sterile lines remained the same throughout the flowering period, and they were unable to satisfy the conditions necessary for self-pollination (**Figures 1B, C**; **Supplementary Figure S2**).

### 3.2 RNA sequencing and quantitative proteomic analysis

To further elucidate the molecular mechanisms responsible for the apparent differences in tobacco stamen development, we conducted RNA-seq and quantitative proteomic analyses. The bud transcriptomes were first sequenced using Illumina technology and six cDNA libraries were constructed, and the RNA-seq raw data were deposited in the Sequence Read Archive (SRA) of the National Center for Biotechnology Information (NCBI) under the accession numbers SRR32473327 to SRR32473332. The raw sequencing data were then processed to remove low-quality reads and the resulting clean reads were mapped to the TN90 reference genome (https://www.ncbi.nlm.nih.gov/datasets/genome/GCF_000715135.1/). The results showed that approximately 406 million reads were successfully aligned with an average alignment rate of 92.02% and a mean GC content of 44.85% per sample (**Supplementary Table S2**), a total of 51,795 genes were identified. Quantitative proteomic data were next obtained from the buds using TMT sequencing, and the raw data were available at the Integrated Proteome Resource Center (https://www.iprox.cn/) under the Project number IPX0011201000. To assess the reliability of the proteomic dataset, quality control analyses were performed, including principal component analysis (PCA) of biological replicates, protein coverage distribution, molecular weight distribution, and unique peptide count. The results were presented in **Supplementary Figures S3**-**S6**. A total of 328,827 secondary spectra, 119,970 validated spectra (PSMs), 62,091 peptides and 10,577 proteins were identified in this analysis.

**Figure 2:**
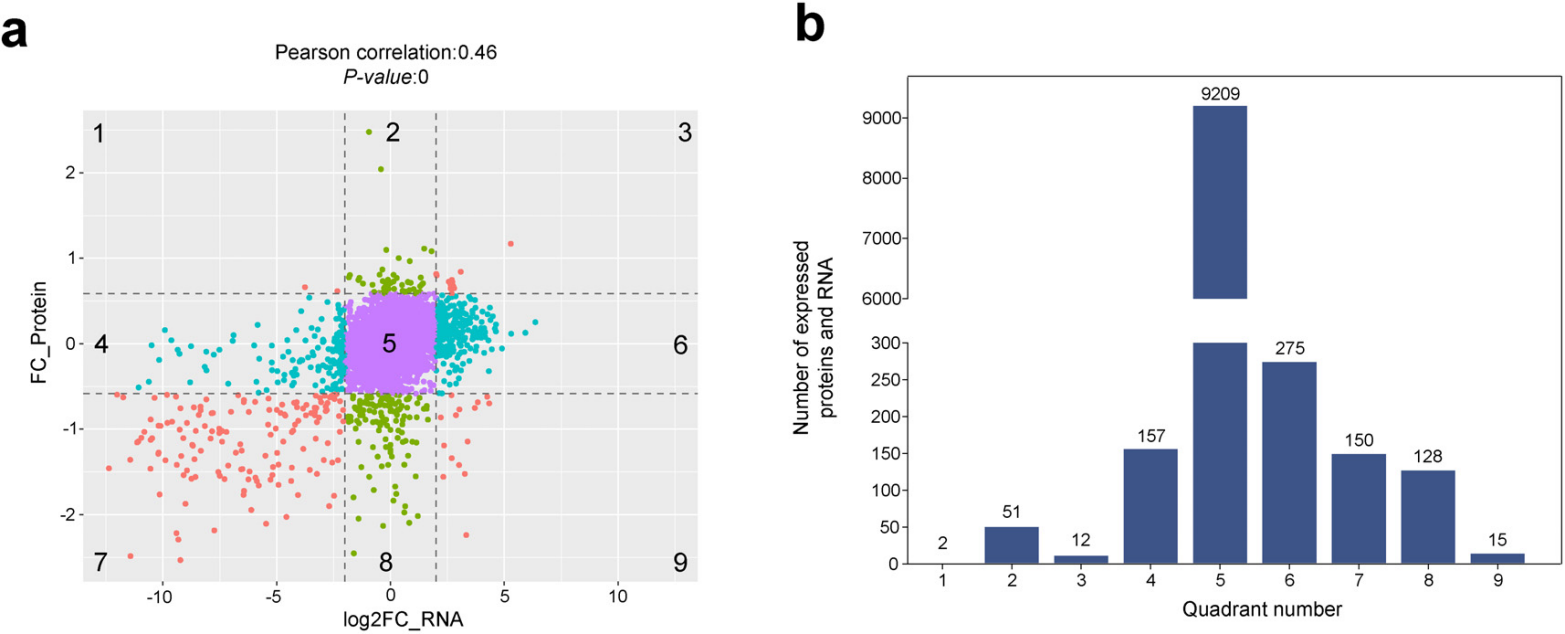
Association analysis between proteins and genes. **(a)** 9-quadrant association plot between genes quantified in the transcriptomic and proteomic datasets. **(b)** Number of genes and proteins enriched in nine quadrants.

To analyse the consistency and variability of the transcriptome and proteome, we performed a nine-quadrant analysis on these datasets to explore the relationship between RNA and protein expression in the sterile and maintainer lines. The results in **Figure 2** revealed that: (1) there was a moderate correlation between RNA and protein expression abundance, with a correlation coefficient of 0.46. (2) Quadrant 3 genes (0.12%) and quadrant 7 genes (1.50%) showed the same expression pattern at both transcriptional and translational levels, whereas quadrant 1 genes (0.02%) and quadrant 9 genes (0.15%) showed the opposite expression pattern at both levels, and quadrant 4 (1.57%) and quadrant 6 genes (2.75%) showed significant differences in expression of the transcript only, while quadrant 2 (0.51%) and quadrant 8 (1.28%) genes showed significant differences in expression of protein only, and no significant differences in expression of any of the quadrant 5 genes (92.10%). Quadrant 4 genes (1.57%) and quadrant 6 genes (2.75%) showed significant differences only in transcript expression, quadrant 2 genes (0.51%) and quadrant 8 genes (1.28%) showed significant differences only in protein expression, and quadrant 5 genes (92.10%) showed no significant changes in expression, so that the percentage of genes with significant changes in expression does not exceed 8%. (3) Considering only the genes with significant changes in expression, the number of genes with single-level expression differences (77.34%, mainly at the transcript level and with significantly larger fold changes in transcript down-regulation) was much higher than the number of genes with differences in expression at both levels (22.66%). (4) For genes differentially expressed at both levels, the same expression pattern (quadrants 3 and 7) prevailed and was mainly down-regulated. These results indicated that the downregulation trend was significant at both the transcriptional and translational levels, with a large fold change observed in the differences.

**Figure 3:**
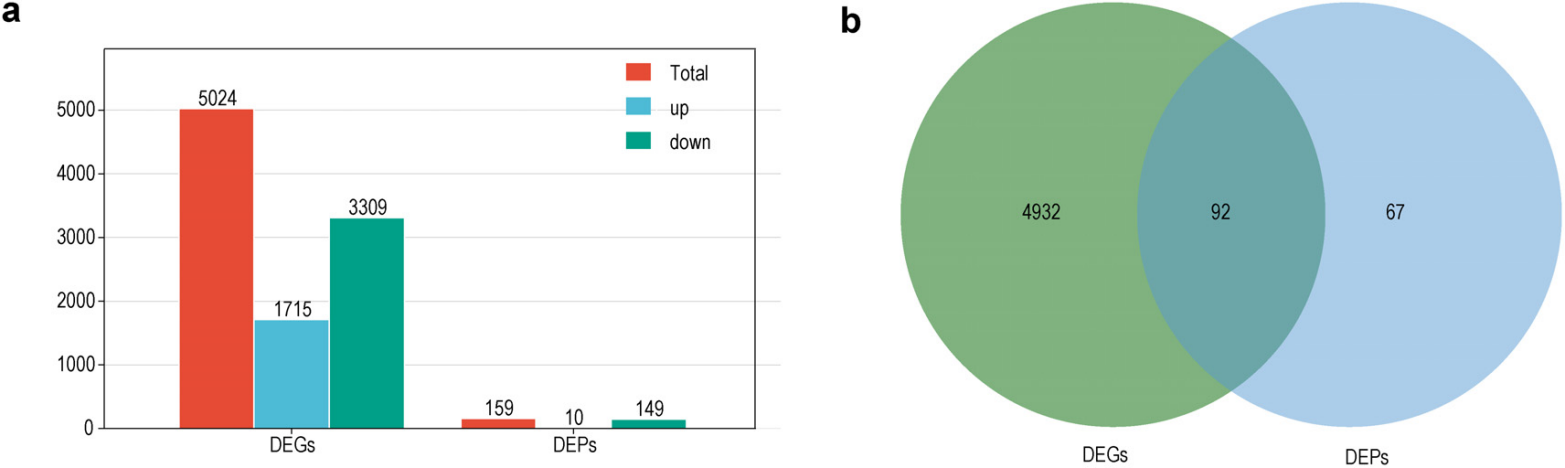
Summary of DEGs and DEPs. **(a)** Number of DEGs and DEPs in the sterile lines; **(b)** Venn diagram of DEGs and DEPs in the sterile lines.

Taken together, a total of 5,024 DEGs (1,715 upregulated and 3,309 downregulated) and 159 DEPs (10 upregulated and 149 downregulated) were identified in the sterile lines as compared to the maintainer lines (**Figure 3a**; **Supplementary Tables S3**, **S4**). It was clear that the number of downregulated DEGs and DEPs was significantly higher than the number of upregulated ones, which results support that most of the CMS-related pathways were downregulated. Among the genes shared by these DEGs and DEPs, up to 87 genes were significantly downregulated at the transcriptional and translational levels, and only 1 gene was significantly up-regulated (**Figure 3b**).

### 3.3 Carbohydrate metabolism was identified as a key pathway by integrated transcriptome and proteome analysis

**Figure 4:**
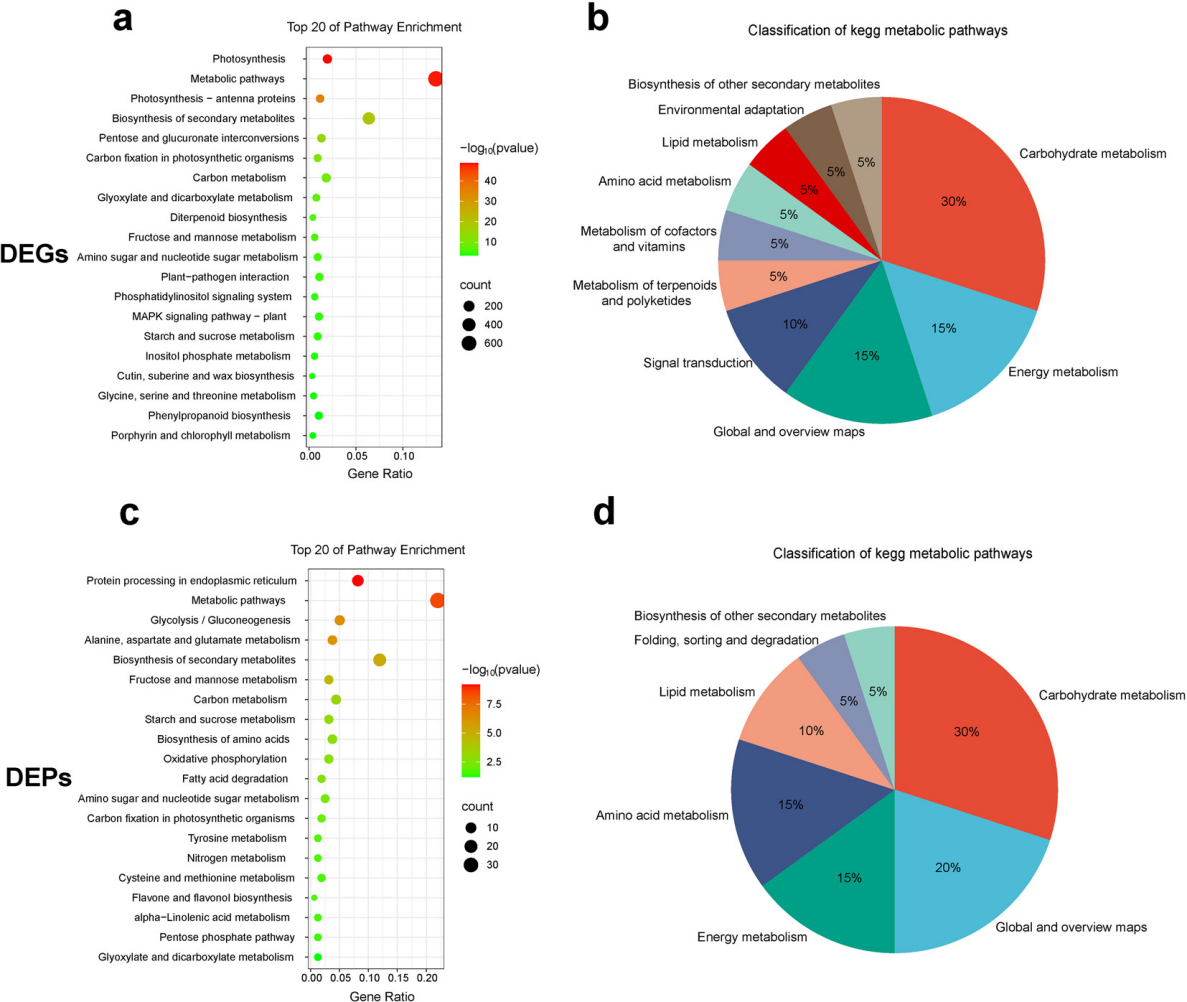
Functional analysis of DEGs and DEPs. **(a, c)** KEGG enrichment analysis of DEGs and DEPs. **(b, d)** KEGG functional classification of DEGs and DEPs.

To further elucidate the major biological functions of the transcriptome and proteome, KEGG enrichment analyses were conducted on DEGs and DEPs, respectively (**Figures 4a, c**). Transcriptomic KEGG analysis revealed that 883 DEGs were involved in the top 20 pathways, ranked by p-value (**Supplementary Table S5**). These pathways were categorized into 10 classes (**Figure 4b**), with 30% involving carbohydrate metabolism. Notably, 228 carbohydrate DEGs accounted for 25.8% of the total enriched genes (**Supplementary Table S6**). Proteomic KEGG analysis revealed that 49 DEPs were involved in the top 20 pathways, ranked by p-value (**Supplementary Table S7**). These pathways were categorized into 7 classes (**Figure 4d**), with 30% involving carbohydrate metabolism. Additionally, 15 carbohydrate DEPs accounted for 30.6% of the total enriched proteins (**Supplementary Table S8**), consistent with the transcriptomic results. Carbohydrate metabolism, including starch and sucrose metabolism, as well as fructose and mannose metabolism, was represented among the top 20 enriched pathways in both the transcriptome and proteome.

### 3.4 Carbohydrate metabolism plays an important role in CMS

**Figure 5:**
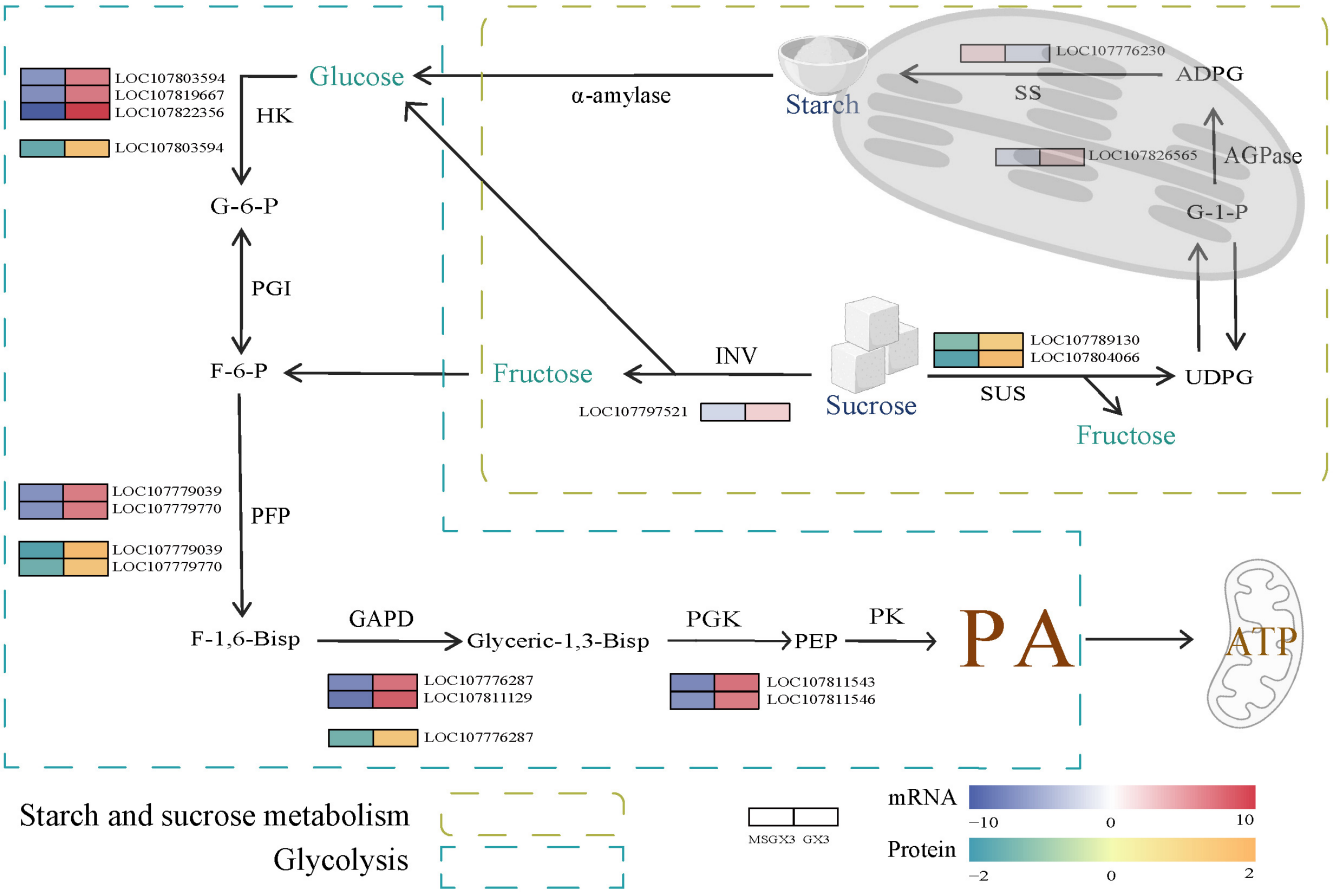
DEGs and DEPs involved in carbohydrate metabolism. Abbreviations are as follows: ADPG—adenosine diphosphate glucose; AGPase—ADP-glucose pyrophosphorylase; F-1,6-Bisp—Fructose-1, 6-bisphosphate; F-6-P—fructose-6-phosphate; GAPD—glyceraldehyde-3-phosphate dehydrogenase; G-1-P—glucose-1-phosphate; G-6-P—glucose-6-phosphate; HK—hexokinase; INV—invertase; PA—pyruvate; PEP—phosphoenolpyruvate; PFP—pyrophosphate-fructose 6-phosphate 1-phosphotransferase; PGI—glucose-phosphate isomerase; PGK—phosphoglycerate kinase; PK—pyruvate kinase; SS—starch synthase; SUS—sucrose synthase; UDPG—uridine diphosphate glucose; α-amylase—Alpha-amylase.

Carbohydrates serve as the material basis for flower buds development and are also key components of the cell wall. **Figure 5** presents the regulatory framework and corresponding expression levels heatmap of DEGs and DEPs associated with the carbohydrate metabolism pathway, comparing tobacco sterile and maintained lines (**Supplementary Table S9**). This pathway produces pyruvate as a respiratory substrate for the TCA cycle, and its aberrant metabolism may impact the synthesis of mitochondrial ATP, resulting in insufficient energy supply during pollen development and ultimately triggering male sterility. During sucrose hydrolysis, the invertase (INV) gene was down-regulated, and the number of proteins in 2-sucrose synthase (SUS) was decreased in sterile lines compared to maintained lines. In sterile lines, during starch synthesis, the gene encoding ADP-glucose pyrophosphorylase (AGPase) was down-regulated, whereas the expression of the gene encoding starch synthase (SS) was up-regulated.

We identified key DEGs and DEPs involved in the glycolytic pathway. The expression and protein levels of a hexokinase (HK) gene were down-regulated and decreased in the sterile lines compared to the maintained lines. Gene expression and protein levels of two pyrophosphate-fructose 6-phosphate 1-phosphotransferase (PFP) genes in sterile lines exhibited varying degrees of down-regulation and decrease, respectively. Both gene expression and protein levels of a glyceraldehyde-3-phosphate dehydrogenase (GAPD) were down-regulated and decreased in sterile lines.

### 3.5 Physiological indices related to carbohydrate metabolism and ATP determination

**Figure 6:**
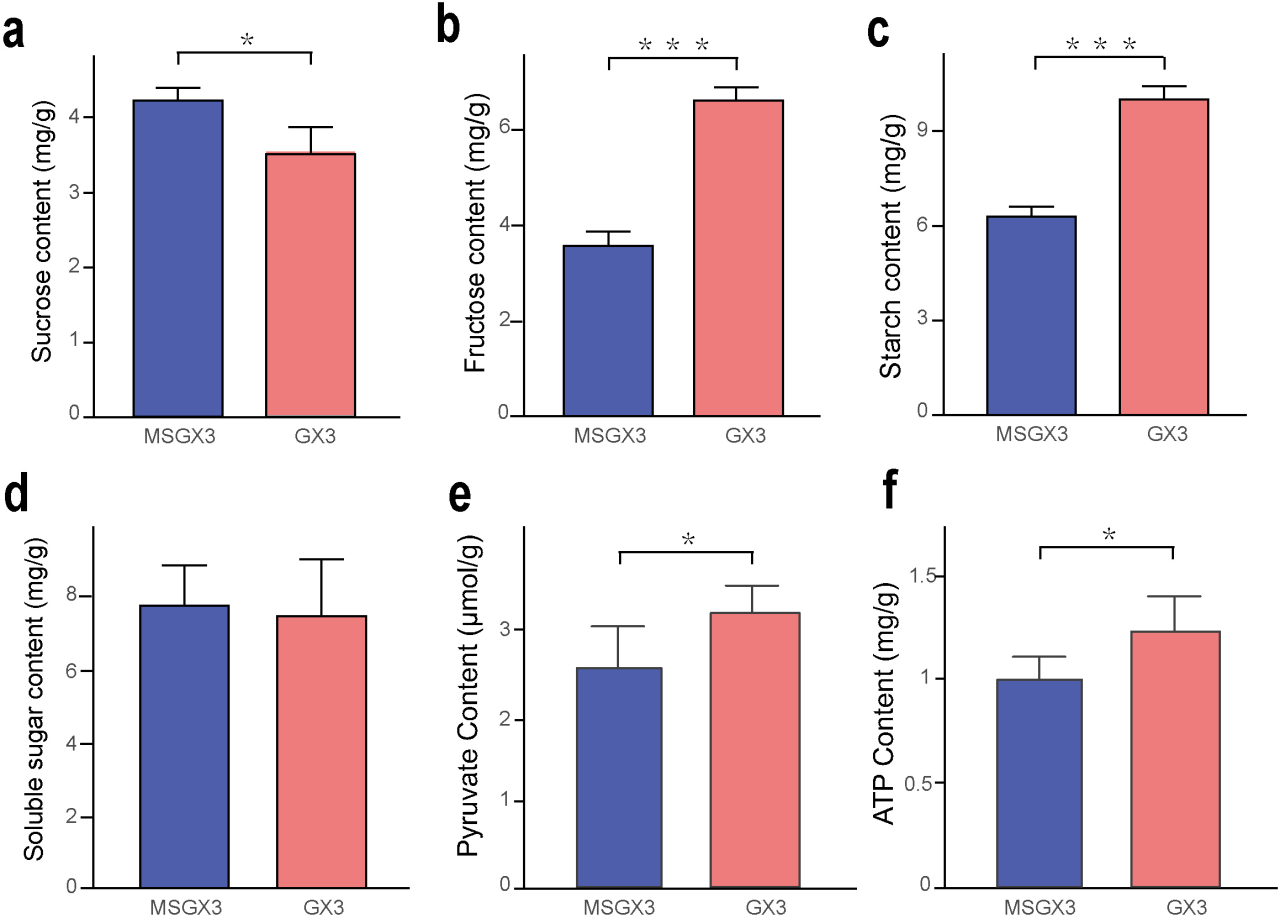
Physiological indices of sterile and maintained lines. **(a)** Sucrose content. **(b)** Fructose content. **(c)** Starch content. **(d)** Soluble sugar content. **(e)** Pyruvate content. **(f)** ATP content. *Significant difference (t-test, 0.01 < p < 0.05); ***Highly significant difference (t-test, p < 0.001).

Gene and protein expression were significantly reduced in sucrose hydrolysis-associated DEGs compared to DEPs in the sterile lines. Therefore, we measured the levels of sucrose, fructose, starch, soluble sugars, pyruvate, and ATP in flower buds of sterile and maintained lines, respectively, to investigate the carbohydrate metabolic pathways involved in CMS in tobacco (**Figure 6**). The results showed that the sucrose content was significantly increased by 20% in the sterile lines compared to the buds of the maintained lines (**Figure 6a**), while the fructose and starch contents were significantly decreased by 86% and 59%, respectively (**Figures 6b, c**); there was no significant difference in the soluble sugar content (**Figure 6d**), and the PA and ATP contents were both significantly decreased by 18% (**Figures 6e, f**). These results indicated that carbohydrate metabolism and ATP content were significantly altered in the sterile lines, consistent with the results of the transcriptomic and proteomic analyses.

### 3.6 Predicted regulatory interactions between transcription factors and carbohydrate metabolism pathways

**Figure 7:**
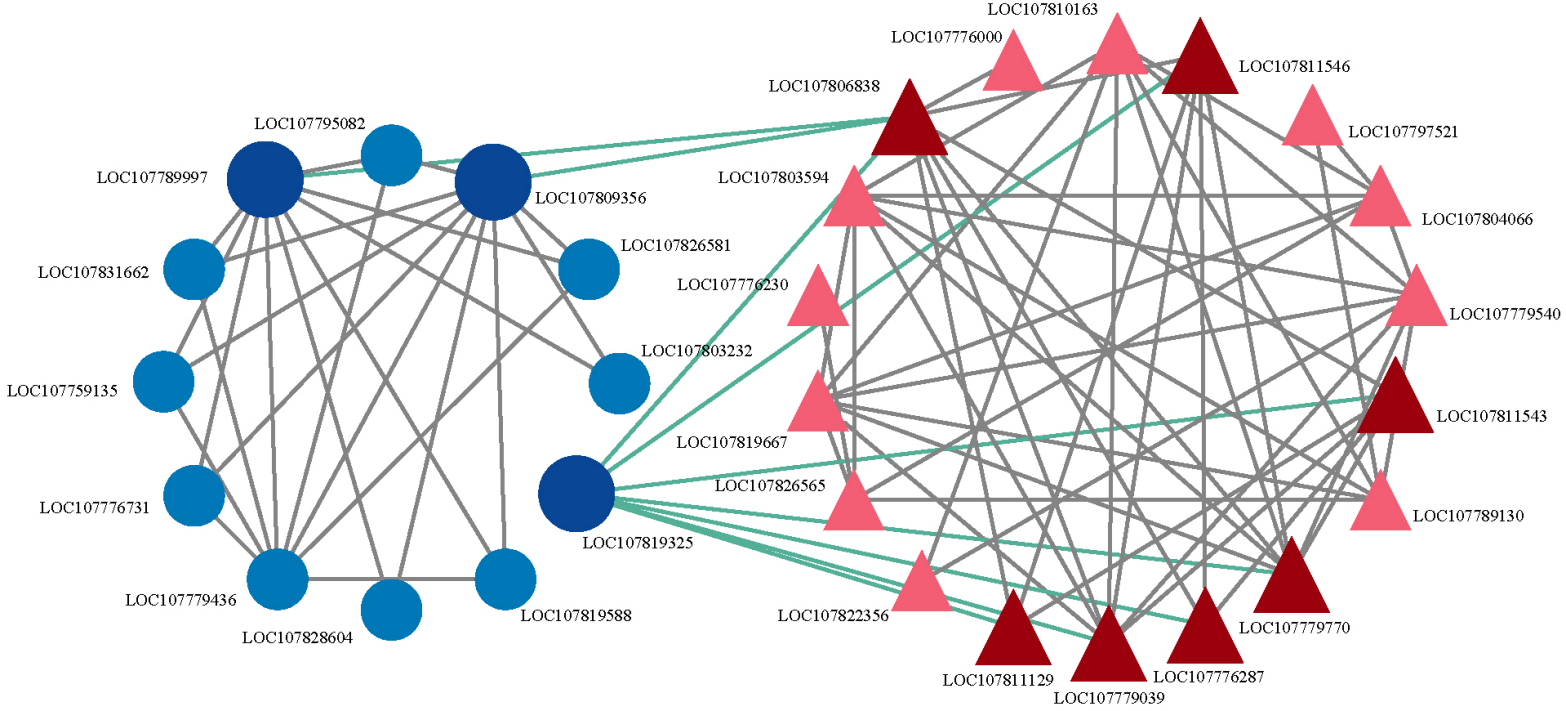
Predicted interaction analysis of candidate genes. Light blue represents transcription factors that do not directly interact with carbohydrates; dark blue represents transcription factors that directly interact with carbohydrate proteins; light red represents carbohydrate proteins that do not directly interact with transcription factors; dark red represents carbohydrate proteins that directly interact with transcription factors.

Transcriptomic and proteomic analyses revealed that CMS in sterile lines is closely associated with carbohydrate metabolism. To further investigate this mechanism, we utilized STRING 10.0 to predict the interactions between transcription factors (TFs) and CMS-related proteins involved in carbohydrate metabolism, and visualized the resulting network with Cytoscape 3.6.1. The analysis suggested that these proteins may form a complex regulatory network with TFs. The majority of predicted key TFs in this network exhibited significant downregulation (**Supplementary Table S10**). The CCCH-type zinc finger protein (C3H) was predicted to interact with phosphopyruvate hydratase (ENO), while the ethylene-responsive transcription factor RAP2.4 was associated with seven carbohydrate-related genes (**Figure 7**). In addition, C3H showed predicted direct connections with the B3 domain-containing transcription factor (B3), LOB domain-containing protein (LOB), and MYB-related transcription factor (MYB), with B3 being the most abundant, followed by LOB.

### 3.7 Putative DEGs related to pollen and tapetum development

By consulting relevant literature and performing database searches, we found a large number of genes associated with tapetum and pollen development in our dataset, most of which were significantly downregulated (**Supplementary Table S11**). Receptor-like kinase (RLK) mediate a complex molecular dialogue among the pollen, tapetum, and middle layer, thereby coordinating male reproductive development (Zhu et al., 2024). In rice, two RLKs, OsTMS10 and its homolog OsTMS10L, redundantly regulate tapetum degeneration and pollen viability in a temperature-dependent manner (Yu et al., 2017). In the present study, nine RLK genes significantly downregulated in MSGX3, suggesting their potential involvement in abnormal tapetum development and pollen abortion. In addition, actin-depolymerizing factors (ADFs) are widely involved in various plant growth and developmental processes, including flowering, pollen development, and pollen tube elongation (Sun et al., 2023). In Arabidopsis, loss of ADF function results in delayed pollen germination and inhibited pollen tube growth (Zhu et al., 2017). In this study, one ADF gene (LOC107783541) was significantly downregulated in MSGX3. They were significantly expressed at the transcriptional level, but not at the protein level, indicating that a complex post-transcriptional regulatory network may exist in MSGX3 to regulate male sterility.

### 3.8 Candidate gene QRT-PCR validation

**Figure 8:**
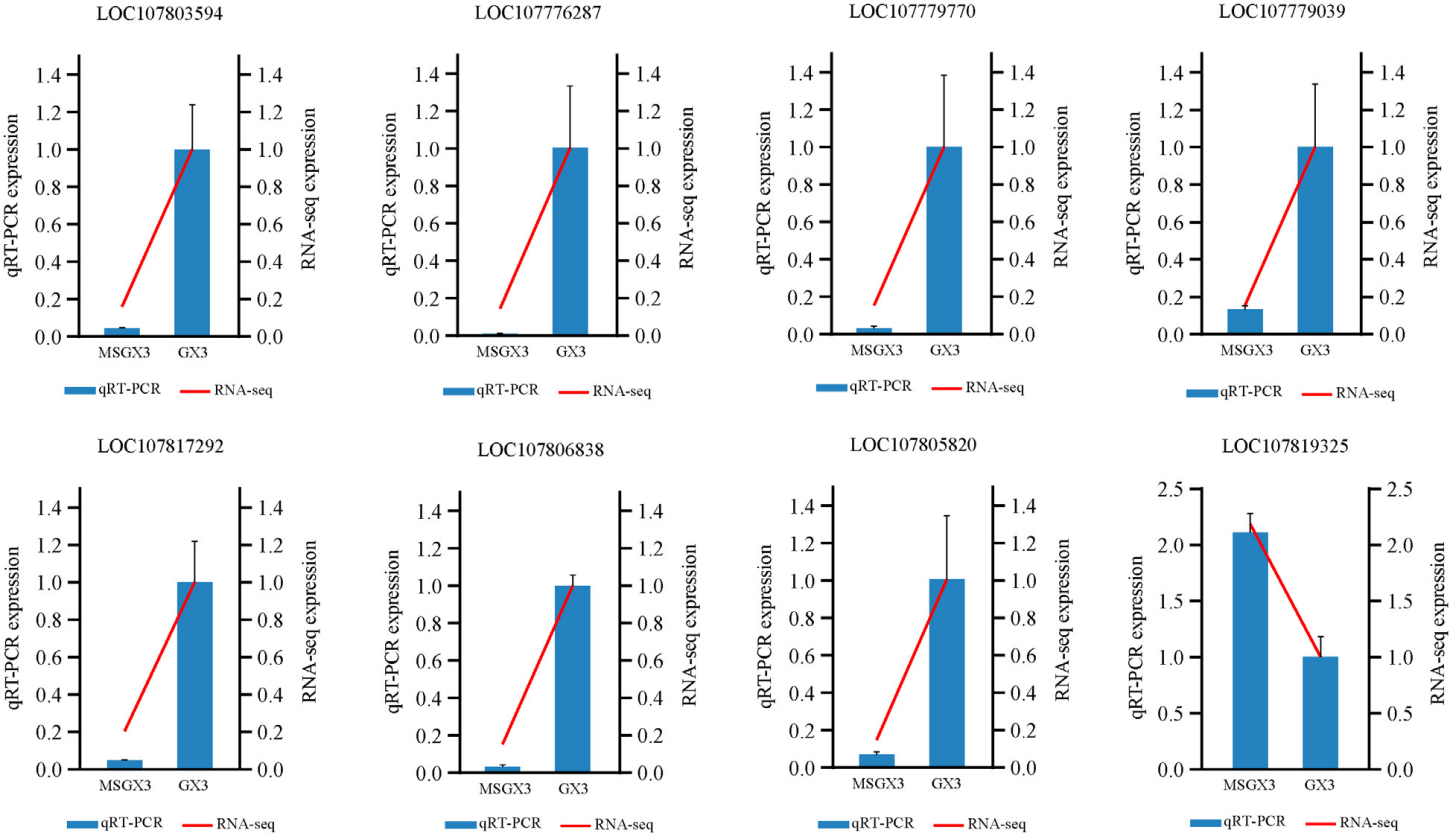
Relative expression of eight genes between tobacco sterile lines and their maintainers detected by real-time quantitative fluorescence PCR.

Eight CMS-associated genes were selected for qRT-PCR analysis to evaluate the quality of the transcriptomic and gene differential expression data, with the Ntubc2 gene used as an internal reference gene for assessing gene expression levels. Based on the relative expression levels, the expression changes of the selected genes were consistent with the patterns observed in the transcriptomic data (**Figure 8**), supporting the reliability of the transcriptomic data and confirming the close relationship of these genes with tobacco CMS.

## 4 Discussion

CMS plants exhibit defective stamen organs that fail to produce fertile pollen, whereas pistil organs remain functional, and the plants are not otherwise defective (Linke and Börner, 2005). The developmental processes of stamens and pollen require large amounts of energy (Shen et al., 2022), with carbohydrates serving as the primary source of energy and carbon for plant growth (Wu et al., 2013). Sucrose and starch are key forms of carbon storage (Smith and Zeeman, 2020), and sucrose catabolism represents one of the largest metabolic fluxes in plants, catalyzed by only two enzymes: *SUS* and *INV* (Barratt et al., 2009). *SUS* catalyzes the reversible conversion of sucrose to uridine diphosphate glucose (UDPG) and fructose, whereas *INV* irreversibly cleaves sucrose into glucose and fructose (Sturm, 1999). Sucrose and its degradation products, glucose and fructose, play critical roles in signaling and regulating plant development (Zhang et al., 2024). Sucrose accumulation is a primary cause of carbohydrate metabolism disorders (Li et al., 2021). Disruption of *INV* typically results in increased sucrose content and abnormal reproductive development, including delayed flowering, impaired male fertility, and abnormal fruit growth (Li et al., 2022). *Arabidopsis INV* knockout mutants have been reported to exhibit impaired vegetative growth phenotypes and prolonged flowering time (Martín et al., 2013). Additionally, *INV* helps provide glucose as a substrate for glycolysis, contributing to ROS homeostasis (Xiang et al., 2011). According to reports, ROS and malondialdehyde (MDA) in plant cell are thought to be important inducible factors of cell apoptosis if excessively accumulated in cells (Li et al., 2015). Increased mitochondrial ROS levels have been observed in Arabidopsis *INV* knockout plants, and elevated ROS may play a role in signaling pathways by triggering the expression response of nuclear genes (Khan et al., 2024). However, excessive ROS accumulation can cause mitochondrial dysfunction, ultimately leading to programmed cell death (PCD) in the tapetum or sporophyte cells (Choudhary et al., 2020). A decrease in SUS enzymes leads to the inability to break down sucrose into UDPG, a key substance in the starch synthesis pathway. Blocked starch synthesis reduces starch content in pollen, ultimately resulting in male sterility (Ba et al., 2022). *SUS* is significantly down-regulated in *Triticum aestivum L.* sterility, resulting in low starch content in pollen grains and excessive ROS accumulation in sterile anthers (Liu H. et al., 2021). Excess reactive oxygen species within plants can be eliminated by relevant enzymes, including superoxide dismutase (SOD), peroxidase (POD), and catalase (CAT) (Hasanuzzaman et al., 2020). During the peak period of anther abortion in CMS cotton, there is an abnormal increase in O_2_, H_2_O_2_, and MDA levels within CMS anthers, coupled with abnormally low activity of SOD, CAT, and POD. This disrupts the balance between ROS production and scavenging, leading to apoptosis of pollen mother cells during this stage (Jiang et al., 2007). In this study, the combined analysis of transcriptomics and proteomics revealed that most of the DEGs and DEPs were involved in carbohydrate metabolic pathways, such as starch and sucrose metabolism, the glycolysis pathway, and fructose and mannose metabolism. We also found that during sucrose hydrolysis, *INV* gene expression and the number of *SUS* proteins were significantly down-regulated and reduced, respectively. The sucrose content in the sterile lines was significantly higher than that in the maintained lines, whereas the fructose and starch contents were significantly lower in the sterile lines. We hypothesized that the down-regulation of *INV* and *SUS* expression in the sterile lines resulted in inefficient sucrose breakdown, which in turn reduced starch synthesis and glycolytic substrate supply. This process led to excessive sucrose accumulation in the sterile lines, along with a significant reduction in starch content. Abnormalities in sucrose metabolism further disrupted ROS homeostasis, ultimately leading to abnormal pollen development.

The glycolytic pathway functions as a metabolic pathway that converts glucose from starch and sucrose metabolism into PA (Giegé et al., 2003). Defects in glucose metabolism and the TCA cycle have been shown to frequently result in male sterility (MS) (Li et al., 2016). In the glycolytic pathway, three key enzymes such as *HK*, *PFP* and *GAPD* play crucial roles in glycolytic metabolism. *HK* catalyzes the first step of glycolysis, and the absence of hexokinase *HK5* in rice has been shown to result in male sterility (Lee et al., 2020). In this study, *HK* (LOC107803594) was significantly downregulated at both the gene and protein levels in sterile lines (**Figure 5**), suggesting a potential role in CMS. *PGI* converts G-6-P derived from HK-catalysed glucose catabolism to F-6-P. *PFP* catalyzes the reversible interconversion of F-6-P and fructose-1,6-bisphosphate (F-1,6-BisP), which is a key step in the regulation of glycolytic metabolic flux (Lim et al., 2009). In rice *PFP* mutants, reduced *PFP* activity leads to impaired carbon metabolism, increased soluble sugar content, and hindered starch biosynthesis (Duan et al., 2016). In this study, *PFP* (LOC107779039, LOC107779770) was significantly downregulated at both the gene and protein levels in sterile lines, with a concomitant reduction in starch content, suggesting that impaired *PFP* expression affects starch synthesis in sterile lines. *GAPD* catalyzes the conversion of F-1,6-BisP to glycerol-1,3-bisphosphate, and studies have demonstrated that *GAPD* expression and catalytic activity in *Arabidopsis* anthers are essential for mature pollen development, with its deletion resulting in male sterility (Muñoz-Bertomeu et al., 2010). In this study, *GAPD* (LOC107776287) was significantly downregulated at both the gene and protein levels in sterile lines, suggesting that reduced *GAPD* activity may be a critical factor contributing to CMS. In addition, PA and ATP levels in sterile lines were significantly lower than those in maintained lines (**Figure 6**), consistent with the findings in MSYY87 (Mo et al., 2023). These results further corroborate that large-scale downregulation of key glycolytic enzymes markedly reduces the supply of PA to the TCA cycle, thereby impairing the mitochondrial respiratory chain. This disruption ultimately results in insufficient ATP synthesis, which may represent a crucial metabolic basis for the occurrence of male sterility.

Flower development is a critical aspect of the reproductive stage in plant development, and TFs have been reported to play essential roles in several biological processes associated with flower buds and pollen development (Jiang et al., 2021). TFs are key components of the regulatory network governing tapetum function and pollen development (Hao et al., 2021). In *Arabidopsis*, five essential TFs involved in anther development and chorion formation constitute the core genetic regulatory pathway (Lou et al., 2018). Our predicted analysis of the transcription factor–carbohydrate proteins interaction network revealed that the transcription factors *C3H* and *RAP2.4* directly interact with carbohydrate-related proteins. In *Arabidopsis*, the *CDM1* gene encoding *C3H* is crucial for regulating callose metabolism and maintaining the integrity of newly formed microspores in male meiotic cells. Defects in the *CDM1* gene have been shown to cause sterility in mutants (Lu et al., 2014).In this study, *C3H* was significantly downregulated in sterile lines, suggesting that this reduction may inhibit microspore development, ultimately leading to CMS. *RAP2.4* belongs to the APETALA2/ethylene response factor (AP2/ERF) family of transcription factors, which are involved in regulating primary and secondary metabolism, controlling growth and developmental processes, and responding to environmental stimuli (Licausi et al., 2013). Rice *RAP2.4* loss-of-function mutants exhibited no significant phenotypic changes, but overexpression of *RAP2.4* led to defects in multiple light and ethylene-regulated developmental processes, including flowering time (Lin et al., 2008). In this study, the *RAP2.4* gene was significantly upregulated, and the encoded protein may regulate pollen development by influencing ethylene-regulated developmental processes. The regenerative meristem (REM) gene family encodes transcription factors belonging to the B3 domain-containing superfamily. Simultaneous knockdown of *REM34*, *REM35*, and *REM36* has been shown to result in partial sterility in *Arabidopsis* (Caselli et al., 2019). Additionally, in *Arabidopsis*, *MYB* mutants exhibit defective anther development, characterized by hypertrophy of the tapetum at the pollen mother cell stage and meiotic microsporidial abortion (Millar and Gubler, 2005). Furthermore, ectopic expression of the *Arabidopsis LOB* gene induces alterations in floral organ size and shape, ultimately causing male sterility (Shuai et al., 2002).

In this study, we identified key factors associated with fertility in tobacco. Morphological observations, transcriptomic and proteomic analyses, physiological and biochemical assays, and bidirectional network analysis revealed that tobacco CMS may be linked to abnormal energy metabolism disorders. Dysregulation of HK, PFP, and GAPD gene expression disrupts levels of the key respiratory substrate PA, leading to reduced ATP synthesis and ultimately inducing male sterility. These findings lay the foundation for systematically understanding the regulatory mechanisms of tobacco floral bud development and their application in hybrid breeding, while also deepening our understanding of floral bud development and male fertility regulation in crops. Nevertheless, it should be noted that the DEPs identified in this study showed lower overlap compared to the DEGs. A low degree of overlap between transcriptomic and proteomic datasets has also been commonly reported in many plant studies (Peng et al., 2015; Yu et al., 2020). Such discrepancies are often attributed to differences in mRNA stability, post-transcriptional regulation, translation efficiency, and protein degradation dynamics (Liu et al., 2016). Further, different data analysis strategies and different bioinformatic models can lead to significantly different results even if the same dataset is being analyzed (Schwanhäusser et al., 2011; Li et al., 2014). In our study, this difference may have been further amplified by the complexity of flower buds, where transcriptional changes can occur in advance of protein-level alterations. Despite this, the overall expression trends of key enzymes involved in carbohydrate metabolism were consistent across both omics levels, supporting the reliability of our integrated findings. Future studies employing higher-depth proteomic analyses, improved protein extraction strategies, or targeted quantitative approaches will further enhance data robustness and provide a more comprehensive view of the molecular regulatory networks underlying CMS.

## Data Availability

All data generated or analyzed during this study are included in this published article and its **Supplementary Information** files. The proteome raw data were available at the Integrated Pro-teome Resource Center (https://www.iprox.cn/) under the Project number IPX0011201000. The RNA-seq raw data were deposited in the Sequence Read Archive (SRA) of the National Center for Biotechnology Information (NCBI) under the accession numbers SRR32473327 to SRR32473332.
