# Supplementary figures and images for: Integrating transcriptomic and proteomic analyses reveals impaired carbohydrate metabolism in tobacco cytoplasmic male sterility
Source: Front Plant Sci. 2025 Nov 19;16:1695628. doi: 10.3389/fpls.2025.1695628 (PMC12672501; doi:10.3389/fpls.2025.1695628)

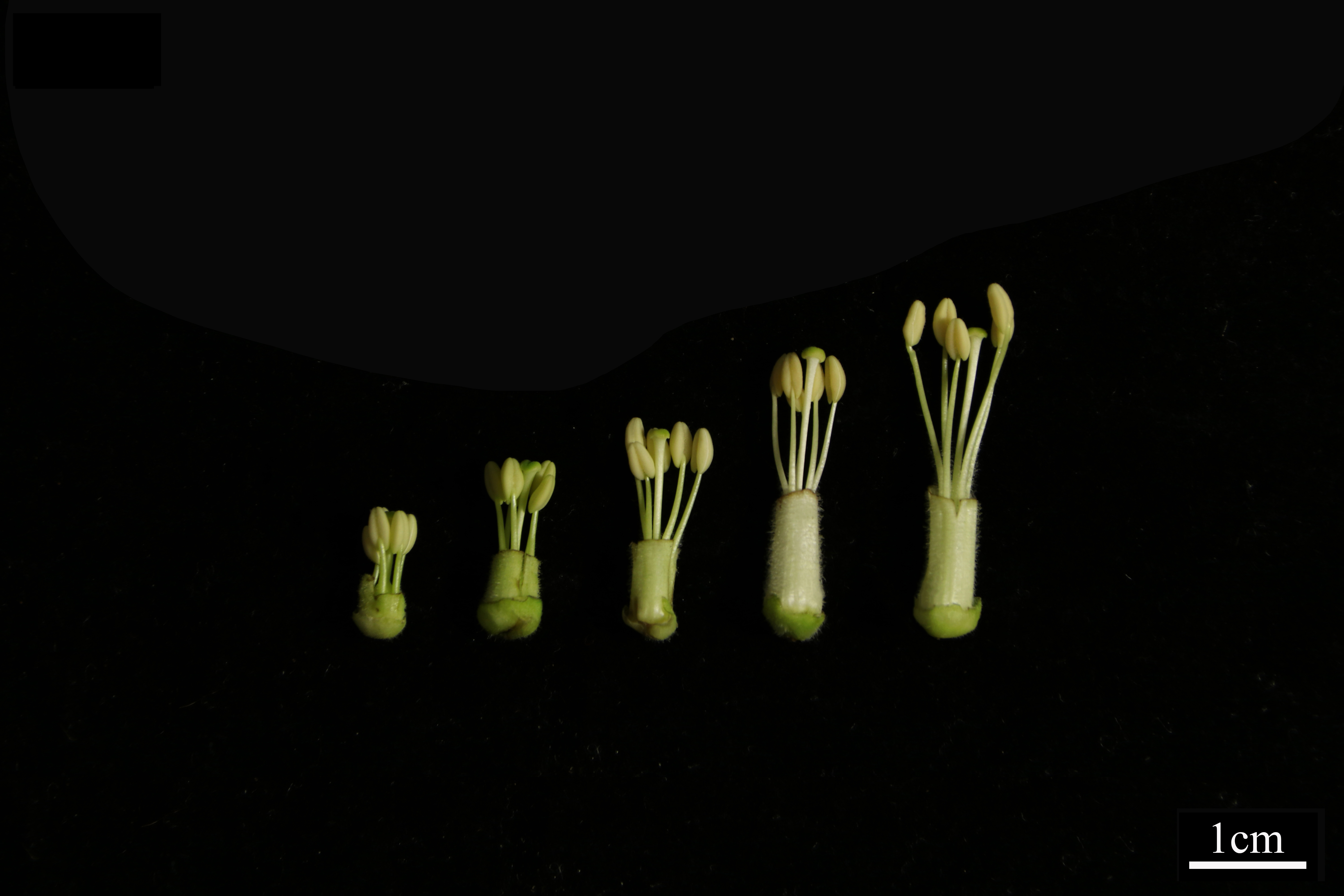

Supplement: Supplementary file 1 [file Image1.jpeg]

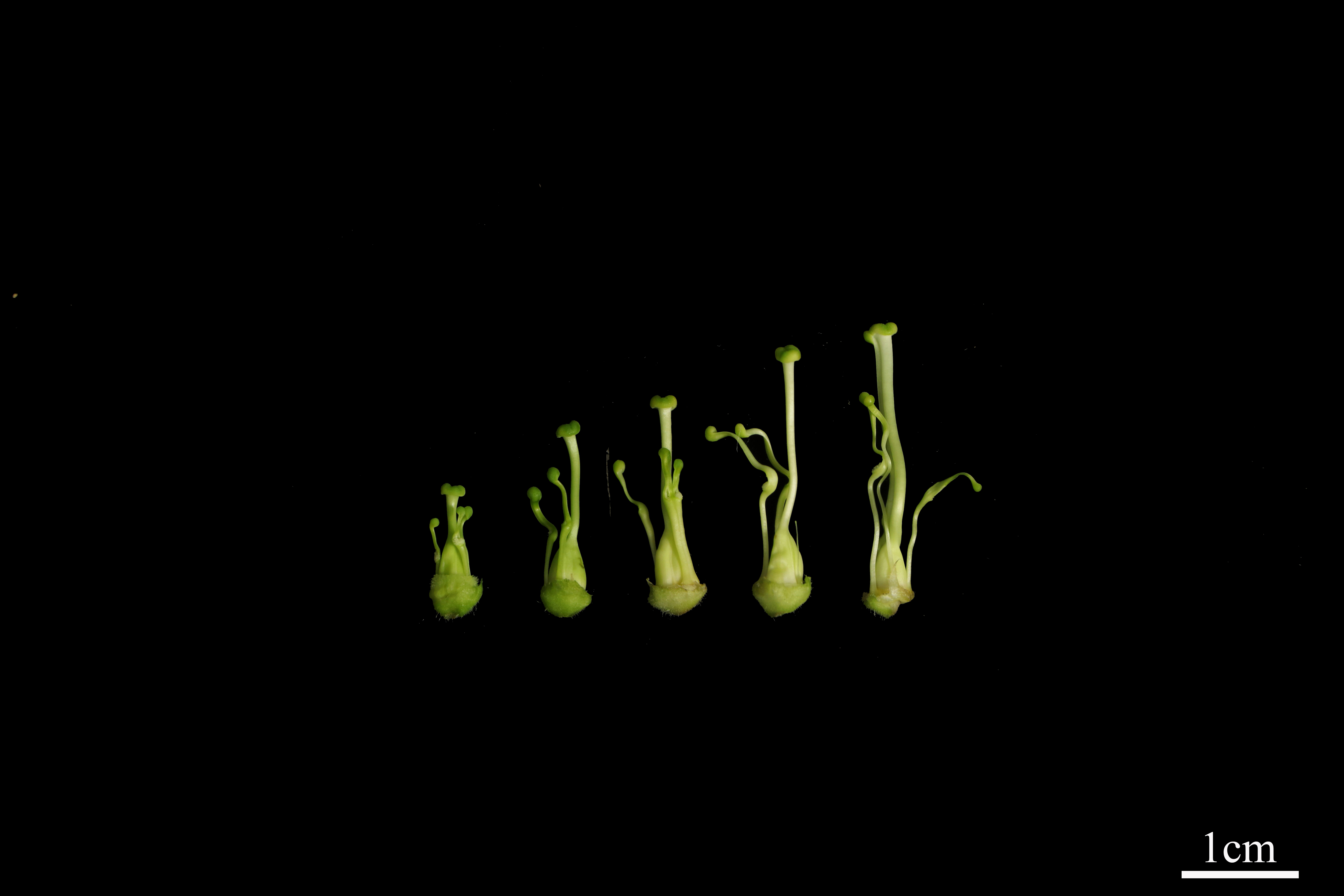

Supplement: Supplementary file 2 [file Image2.jpeg]

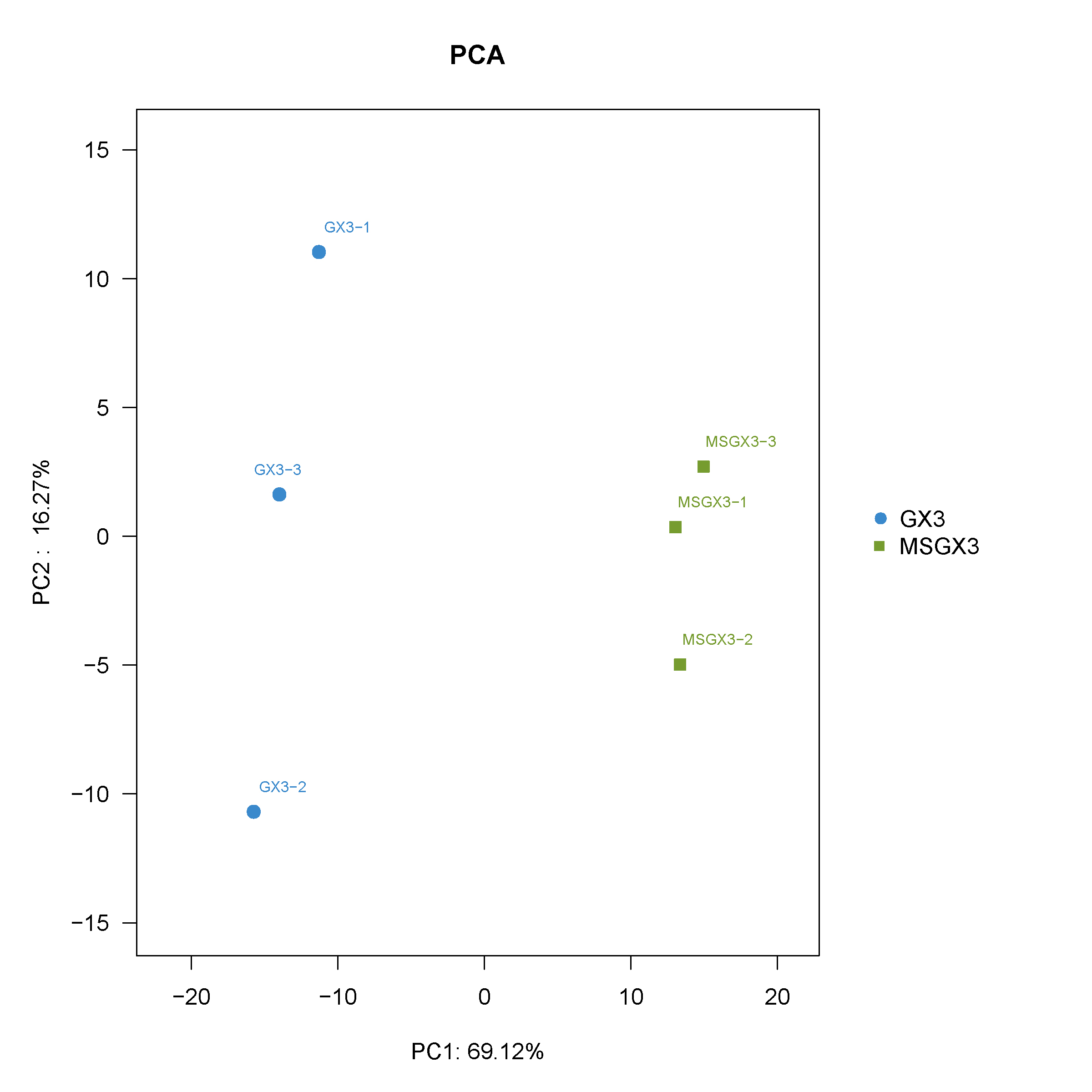

Supplement: Supplementary file 3 [file Image3.jpeg]

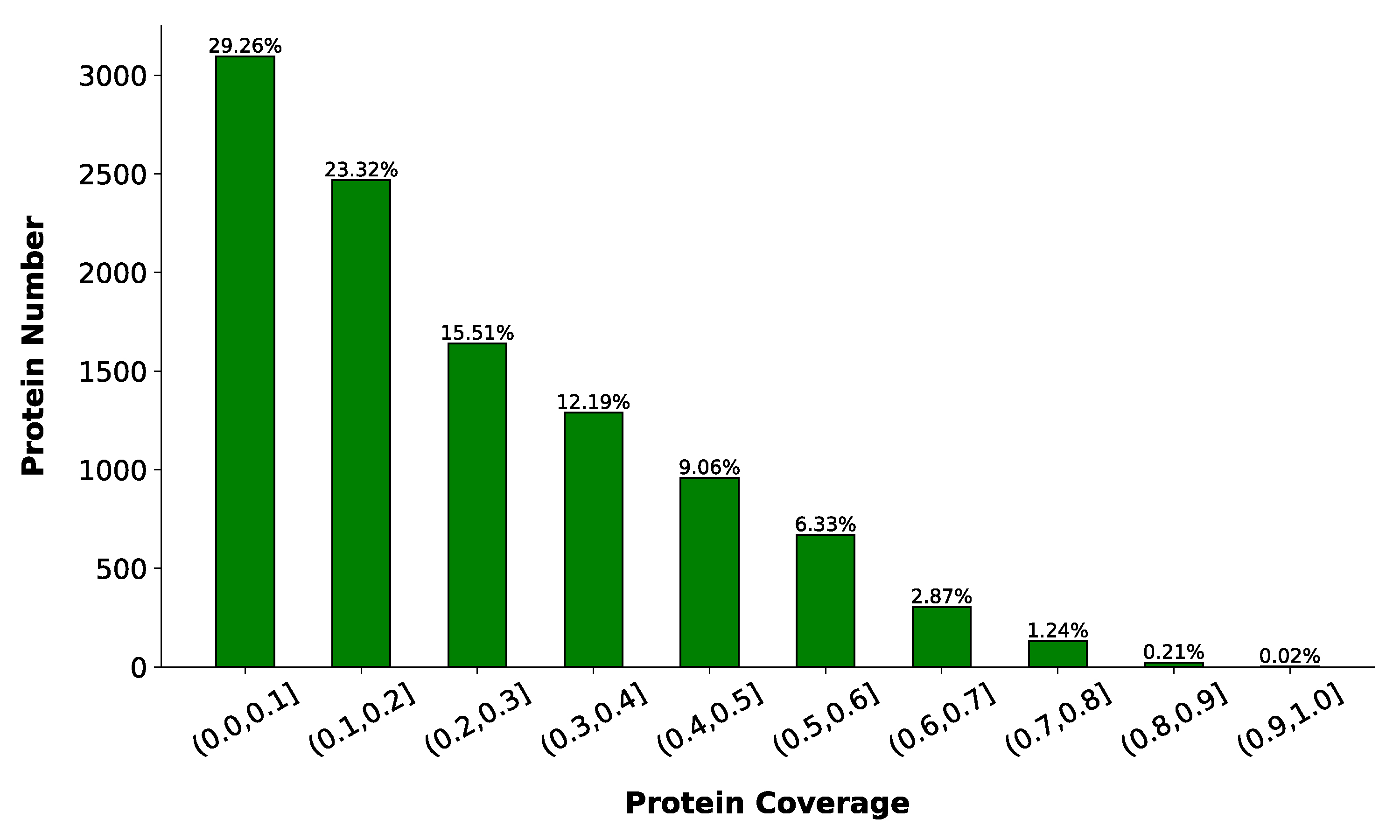

Supplement: Supplementary file 4 [file Image4.jpeg]

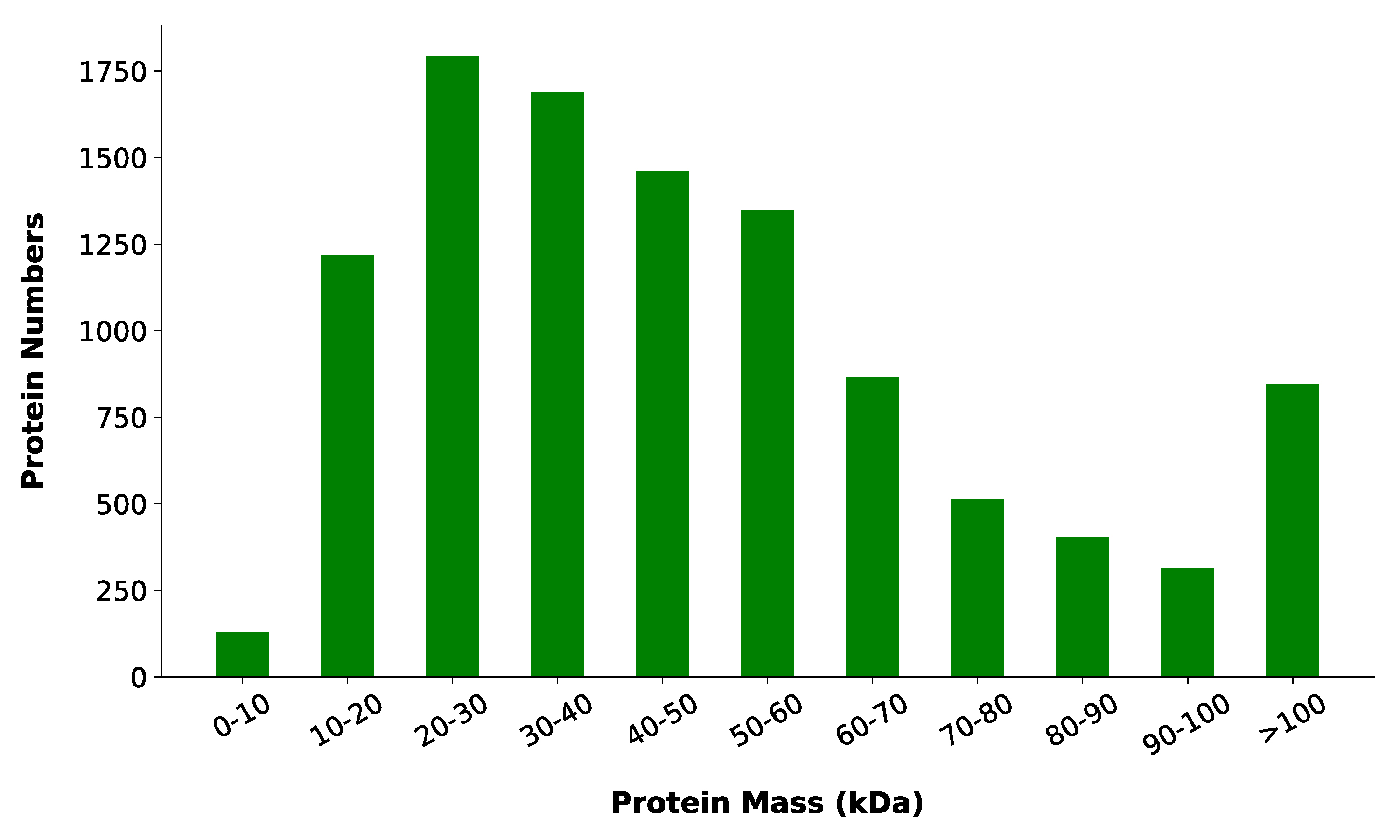

Supplement: Supplementary file 5 [file Image5.jpeg]

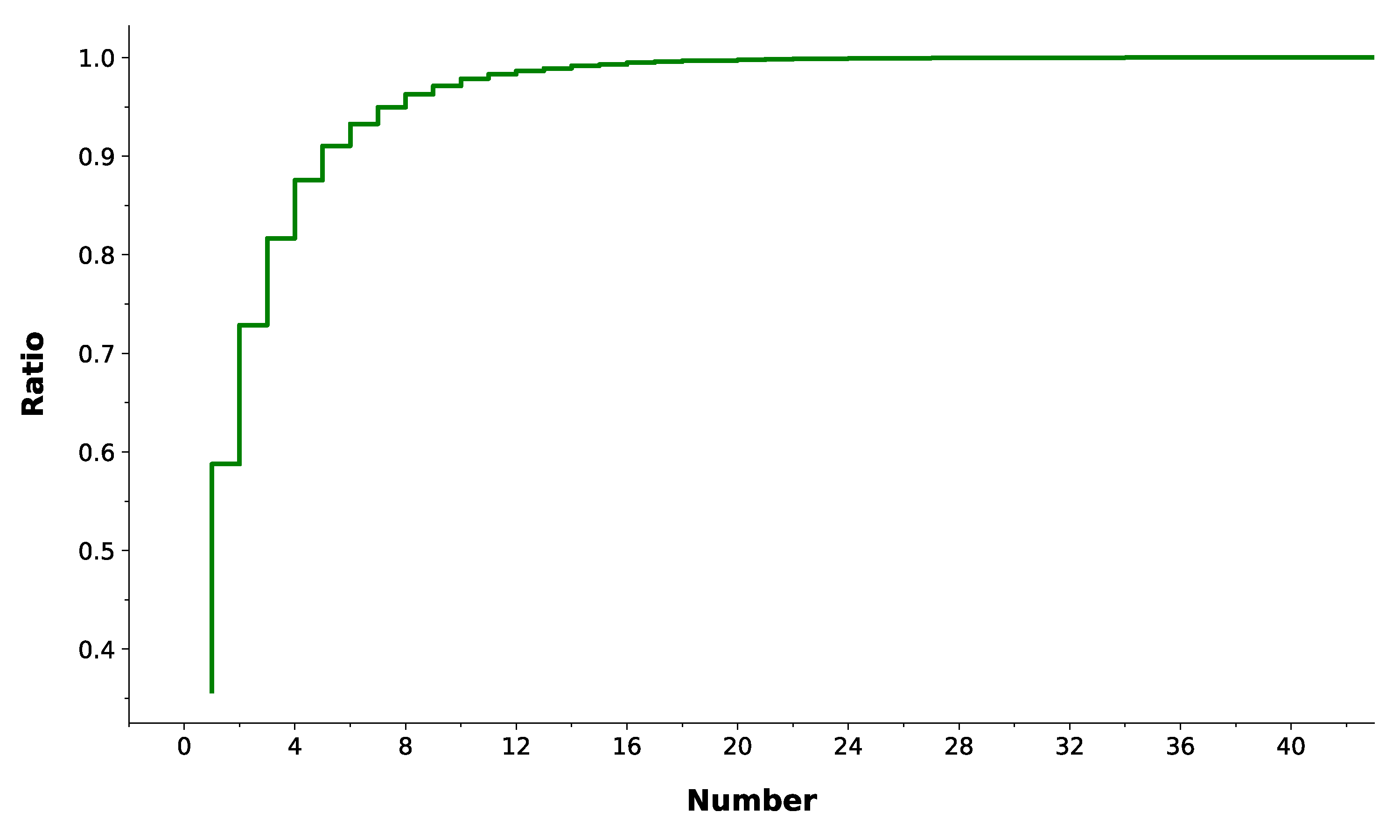

Supplement: Supplementary file 6 [file Image6.jpeg]
